# Application of integrated Korean forest growth dynamics model to meet NDC target by considering forest management scenarios and budget

**DOI:** 10.1186/s13021-022-00208-8

**Published:** 2022-05-23

**Authors:** Mina Hong, Cholho Song, Moonil Kim, Jiwon Kim, Sle-gee Lee, Chul-Hee Lim, Kijong Cho, Yowhan Son, Woo-Kyun Lee

**Affiliations:** 1 Department of Environmental Science and Ecological Engineering, Korea University, Seoul, 02841 Republic of Korea; 2 OJEong Resilience Institute (OJERI), Korea University, Seoul, 02841 Republic of Korea; 3 Department of ICT-Integrated Environment, Pyeongtaek University, Pyeongtaek, 17869 Republic of Korea; 4 Forest Resource Research Center, Chonnam National University, Gwangju, 61186 Republic of Korea; 5 College of General Education, Kookmin University, Seoul, 02707 Republic of Korea

**Keywords:** Forest growth model, Forest management, CO_2_ sequestration, NDC, Forest budget, Climate change

## Abstract

**Background:**

Forests are atmospheric carbon sinks, whose natural growth can contribute to climate change mitigation. However, they are also affected by climate change and various other phenomena, for example, the low growth of coniferous forests currently reported globally, including in the Republic of Korea. In response to the implementation of the Paris Agreement, the Korean government has proposed 2030 greenhouse gas roadmap to achieve a Nationally Determined Contribution (NDC), and the forest sector set a sequestration target of 26 million tons by 2030. In this study, the Korean forest growth model (KO-G-Dynamic model) was used to analyze various climate change and forest management scenarios and their capacity to address the NDC targets. A 2050 climate change adaptation strategy is suggested based on forest growth and CO_2_ sequestration.

**Results:**

Forest growth was predicted to gradually decline, and CO_2_ sequestration was predicted to reach 23 million tons per year in 2050 if current climate and conditions are maintained. According to the model, sequestrations of 33 million tCO_2_ year^−1^ in 2030 and 27 million tCO_2_ year^−1^ in 2050 can be achieved if ideal forest management is implemented. It was also estimated that the current forest management budget of 317 billion KRW (264 million USD) should be twice as large at 722 billion KRW (602 million USD) in the 2030s and 618 billion KRW (516 million USD) in the 2050s to achieve NDC targets.

**Conclusions:**

The growth trend in Korea's forests transitions from young-matured stands to over-mature forests. The presented model-based forest management plans are an appropriate response and can increase the capacity of Korea to achieve its NDC targets. Such a modeling can help the forestry sector develop plans and policies for climate change adaptation.

## Background

Climate change is considered a global issue that affects our lives, surrounding environment, and socioeconomic sectors [1]. The Paris Agreement is proposed as a “new climate regime”, replacing the Kyoto Protocol that expired at the end of 2020 [2]. It includes long-term goals for greenhouse gas (GHG) reduction, introduction of market mechanisms, climate change adaptations, implementation checks, and technology transfers [3]. The proposition of the “new climate regime” with the Paris Agreement will result in active international efforts to reduce GHG emissions [4]. After the adoption of the Paris Agreement, each country is expected to submit Nationally Determined Contributions (NDCs) and voluntarily set goals and plans that also address various cross-cutting issues [5]. Among them, the possibility of carbon emission reduction was reviewed sector-by-sector (e.g., industries, and energy), and the Paris Agreement emphasized the necessity of maintaining and promoting carbon sinks, including forests [6].

In response, the NDC submitted by each country should also include the utilization of carbon stocks and the sequestration functions of forests [7]. Accordingly, the Republic of Korea has increased its interest and investment in carbon reduction activities and is promoting carbon sequestration functions through forests, to achieve their NDC target in accordance with the 2030 Greenhouse Gas Roadmap [8]. According to the National Institute of Forest Science, current net amount of CO_2_ sequestration of the forest sector is estimated to be approximately 45 million tons per year but the expected amount of CO_2_ sequestration is 13 million tons per year in 2050 [9]. Therefore, the Korean government has set a CO_2_ sequestration target of 26 million tons by 2030 through the promotion of forest management and accelerated preparation [10], under the “2050 Carbon Sequestration and Long Term Strategy” [11]. The 2050 GHG reduction strategy is currently being reviewed and will upgrade the forest GHG inventory plans, including afforestation, reforestation, forest tending, roundwood production and supply expansion, and unused biomass [12].

Carbon sequestration will have to be expanded to meet the NDC for the forestry sector. This will also require enhancing the forest health, in accordance with proper forest management practices in changing climatic conditions. In addition to mitigating global warming, afforestation and efficient forest management will provide various public service functions, such as ecosystem conservation, water retention capacity, air purification, prevention of soil erosion, and provision of recreational functions [13]. In the Republic of Korea, forests comprise approximately 63% of the land, which can be considered a significant carbon sink [14–16]. Consequently, the Republic of Korea emphasizes the importance of forest management strategies [17]. This requires synthetic considerations that have not been considered in previous investigations [18]. Hence, this study sought to predict these synthetic considerations using a model [19].

Although Korea’s forest policy previously focused on the development of forest resources for erosion control and greening, it later changed to a systematic sustainable forest management objective [20]. Therefore, Korea's forest management approach, from the past to the present, has primarily contributed to forestry governance and economic development, as it is implemented under state-based policies [21, 22]. In particular, the Korea Forest Service is responsible for forest GHG evaluation, and it executes the actual forest budget and establishes the forest unit price [23]. However, discussions regarding forest management measures that can promote carbon sinks are relatively inactive [24]. Furthermore, in previous studies, only thinning or cutting of forests was simulated, and dynamic growth was only modeled for individual tree species or the entire stand, depending on the future climate change scenario [25, 26]. It was previously difficult to understand which forest management strategies were advantageous and to identify the extent of management required to achieve the forest areas and yields set as policy targets. However, current modeling technologies can be used to address this problem by analyzing valid policy scenarios for NDC achievement.

Therefore, in this study, a spatiotemporal dynamic stand-growth model capable of reflecting forest growth mechanisms under climate change was used to analyze growth trends and determine the amount of CO_2_ sequestration for a range of forest management scenarios. This study will not only contribute to the achievement of the NDC, but also improve the effectiveness of the contribution through economic analysis.

## Methods

### Study area

**Fig. 1:**
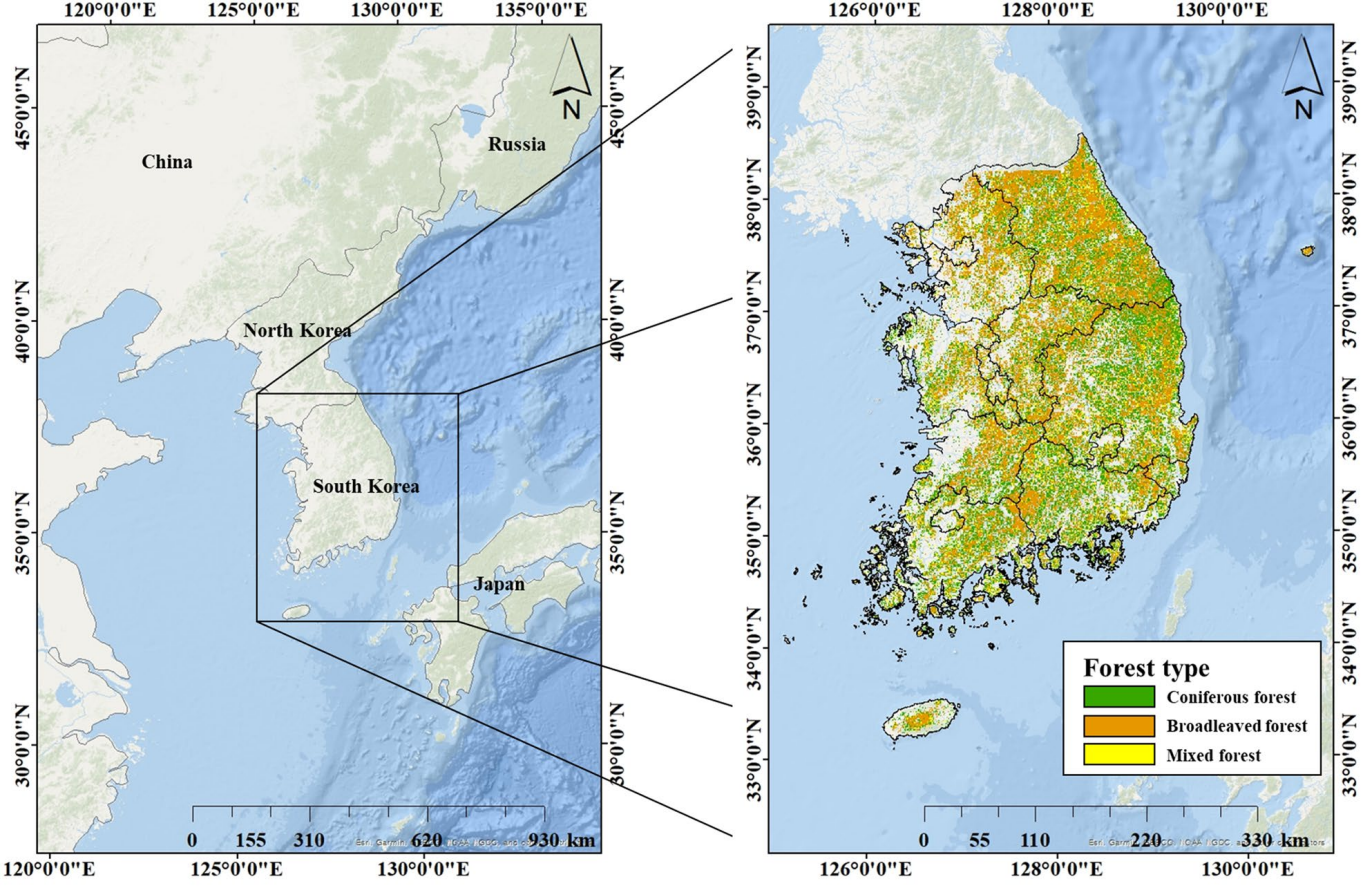
Study area and forest types (2018) as reported by the Korean Forest Service

The study area comprised the forest area in the Republic of Korea (longitude: 124° 54–131° 6 and latitude: 33° 9–38° 45). Korean forests include coniferous forests (mainly *Pinus densiflora*), deciduous broad-leaved forests (mainly *Quercus* spp.), and mixed forests, comprising approximately 37%, 32%, and 26%, respectively, of the total forest area in 2018 [27–29] (Fig. 1). They currently cover 62.6% (6.28 million ha) of Korean land; however, in the 1950s, they only covered 35% [30, 31]. They can be regarded as successful examples of forest restoration utilizing good management practices. However, the restored forests are currently experiencing management challenges [32]. As many stands in the Korean forests are becoming old, they suffer risks such as the decreasing growth of conifers due to climate change [33–35]. The government implements forest management policy through the 6^th^ basic forest plan [36]. This study analyzed the ability of Korean forests to effectively cope with anticipated climate change.

### NDC assessments in the forestry sector

#### Dynamic stand-growth model

**Fig. 2:**
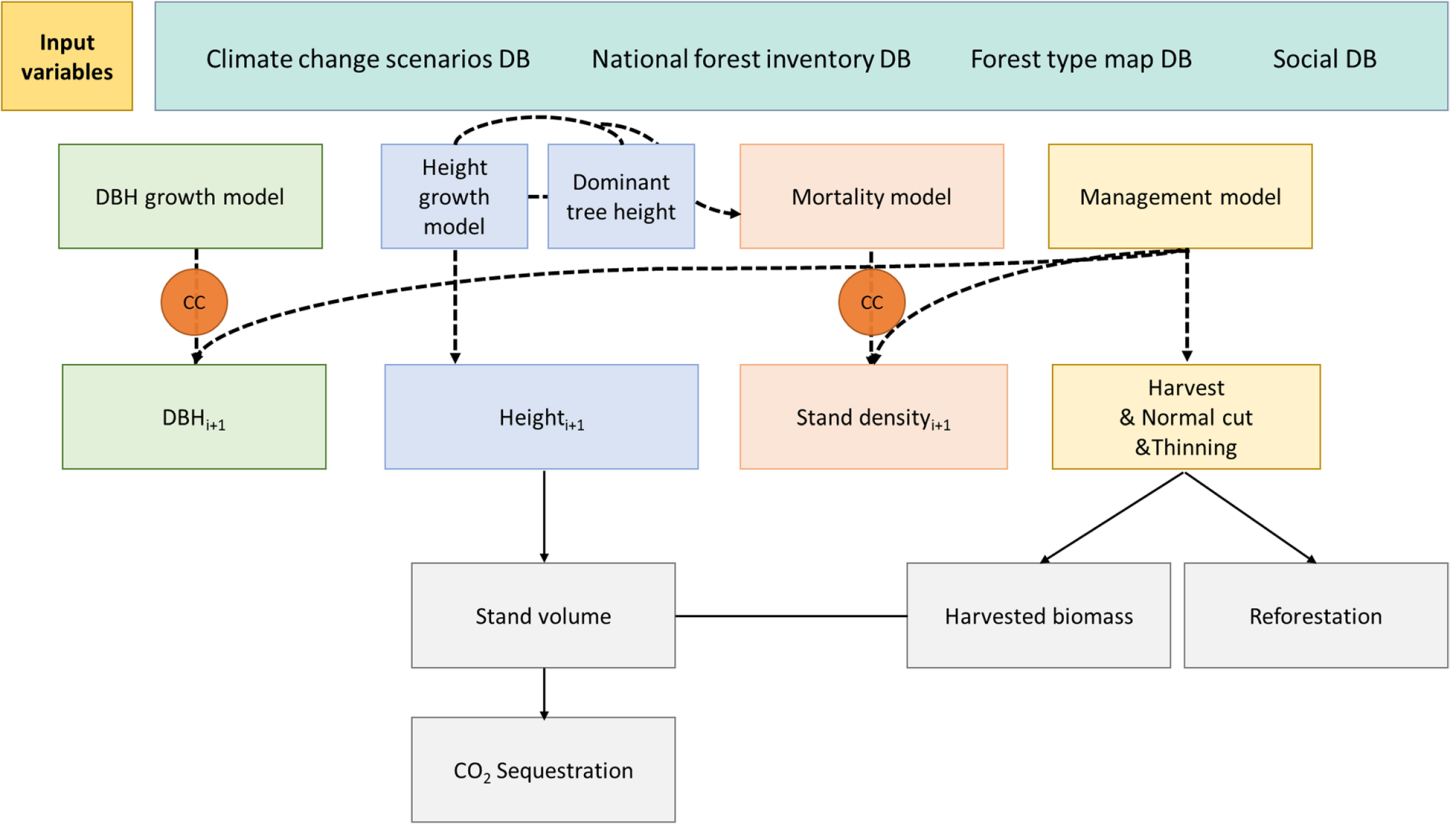
Schematic workflow of the KO-G-Dynamic model processes. Current information on the forest physiognomy was based on the Korean National Forest Inventory, map of forest type, topography, and social data. Subsequently, the site index and stand density were estimated using the stand age and dominant tree information. Then, the mean stand diameter at breast height could be estimated. The stand volume and C stock were estimated using the mean diameter at breast height, tree height, and stand density. To predict future forest physiognomy, the diameter at breast height and stand density were derived using a climatic change scenario (CC), and stand volume, C stock, and CO_2_ sequestration were calculated. The forest management decision-making algorithm assumes that the stand volume, applied with silvicultural interventions, is converted to wood and logging residues. It is assumed that harvested forest areas are reforested with the appropriate tree species to address climate change

In this study, the “KO-G-Dynamic model” a dynamic stand-growth model, was used to evaluate the CO_2_ sequestration as aimed in the NDC [28] (Fig. 2). The KO-G-Dynamic model has previously helped enhance the forest growth model [37–40]. It can accurately predict the growth of temperate forests (which grow yearly), also considering the climatic impacts overlooked by traditional dynamic growth models [28, 41]. These climate factors affect diameter at breast height (DBH) and mortality [39, 40, 42]. This reduces the uncertainty in future growth estimates under climate change, facilitating the assessment of vulnerable forest areas according to Korea’s ecological and environmental characteristics [37, 40]. For this study, Korean forests were divided into seven major types: red pine (*Pinus densiflora*), Japanese larch (*Larix kaempferi*), Korean pine (*Pinus koraiensis*), cork oak (*Quercus variabilis*), Mongolian oak (*Quercus mongolica*), mixed forest A (Mixed-A: red pine and cork oak), and mixed forest B (Mixed-B: red pine and Mongolian oak), based on mapped forest type classification. These results can then be used to derive forest growing stock volume using information such as the diameter at breast height, tree height, and stand density (Eq. (1)).

1 $${\mathrm{V}}_{ij}=a\cdot  DB{H}_{ij}^{b} \cdot  H{m}_{ij}^{c}\times {N}_{ij}$$

where $i$ is the serial number for each stand, $j$ is the year, DBH is the stand average diameter at breast height, Hm is the average tree height of the stand, and N is the stand density (trees/ha).

Coefficients (a, b, and c), determined for each tree species (Table 1), were incorporated with the biomass allometric equation data developed by the National Institute of Forest Science (NIFoS) [43]. The DBH was estimated using the forest growth model (Eq. (2)), Hm was calculated using the site index and the age of stand, Age was calculated based on the age-class of the vector-based forest map which classified forest age by every 10 years, compared with the age data of the NFI. Therefore, raster data set had averaged data following previous studies [28], and Nha was analyzed using a tree mortality model.

**Table 1:** Coefficients used to calculate the forest growing stock volume by tree species (NIFoS, 2010)

Coefficient	*Pinus densiflora*	*Larix kaempferi*	*Pinus koraiensis*	*Quercus variabilis*	*Quercus mongolica*
a	0.000201	0.000088	0.000015	0.000047	0.000344
b	1.7593	1.7828	2.4239	1.8603	1.3639
c	0.6583	0.9397	0.8651	1.0589	0.879

2 $$\mathrm{DBH}=\mathrm{f}\left(\mathrm{Age},\mathrm{ SI},\mathrm{ Nha}\right)$$

where SI is the site index, Nha is the stand density (trees/ha), and Age is the age of the stand.

The mortality rates of stands can be obtained using the relative density index, which is derived from the maximum stem number determined by Sterba (1987) [44]. The DBH development formula was used to derive an equation for the estimation of maximum stand density based on the mean of the dominant tree height and the mean of the DBH. Kim et al. [39] not only identified the relationship between the estimated maximum and actual stand density ratio, but also mathematically analyzed the relationship between maximum stand density reduction and the actual mortality (Eq. (3)).

$$\left(\frac{{Mortality}_{i}}{{Nmax}_{i} - {Nmax}_{i+1}}\right)= {a\cdot  e}^{b\cdot  \left(\frac{{Nha}_{i}}{{Nmax}_{i}}\right)}$$

3 $${Mortality}_{i}=a\cdot  {e}^{b\left(\frac{{Nha}_{i}}{{Nmax}_{i}}\right)}\cdot  ({Nmax}_{i} - {Nmax}_{i+1})$$

where ${Mortality}_{i}$ is the actual mortality at stand age $i$, ${Nha}_{i}$ is the stand density at stand age $i$, and ${Nmax}_{i}$ is the maximum stand density at stand age $i$.

For forest management assessment, the area of reforestation and the area of management with maximum forest growth were estimated in manageable areas [30]. It was assumed that the legal final cut would be performed among tree species as currently announced by the Korea Forest Service [45]. The forest management area was fixed for each scenario, and the stand with the highest stand age was cut first (Eq. (4)). If the forest physiognomy of the same age class is greater than the number of potential cutting areas within the size of the potential cutting area determined for each scenario, the forest growing stock volume is cut in order of highest to lowest. Crown thinning was implemented twice a year [46–48].

4 $${PH}_{i}= \sum [\left({Age}_{kij}\ge {FA}_{j}\right)\cap \left(MA\right)]$$

where ${PH}_{i}$ is the potential cutting area for year $i,$ ${Age}_{kij}$ is the $k$ stand, $i$ is the year, $j$ is the stand age for the tree species, ${FA}_{j}$ is the legal final cutting age of tree species $j$, and MA is the area where forest management is possible.

In the 5^th^ national forest inventory, the site index for each stand was estimated and applied to the model based on the dominant tree height information from the enumeration districts. It was calibrated based on further data from the 6^th^ national forest inventory. Using the above formulas, the accumulation and growth volumes of forests can be analyzed annually [28, 39].

#### CO_2_ sequestration in forests

In this study, the amount of CO_2_ sequestration was calculated for seven dominant forest types in Korea to determine the feasibility of sequestering 26 million tons by the 2030s, as proposed in the basic roadmap for GHG reduction.

For this calculation, the calculation method of CO_2_ sequestration basically utilizes the IPCC 2006 guidance provisions method and follows the stock change method of the forest land part [49, 50]. The National Institute of Forest Science which analyzes forest carbon in South Korea also uses the methodology to calculate CO_2_ sequestration [43]. At this time, it is estimated through sequestration factor for each tree species based on the growth according to the annual difference in stand volume. In this study, carbon stock (*C*) was calculated by using country-specific factors such as basic wood density (*D*), biomass expansion factor (BEF), and root/shoot ratio (*RS*) developed by the National Institute of Forest Science in the stand volume calculated through the growth model [28] (Table 2, Eq. 5). This estimated the annual change in carbon stock (*CS*) in remaining forest land (Eq. 6) and converted to CO_2_ sequestration (Eq. 7).

**Table 2:** Carbon emission factors for the major Korean tree species (NIFoS, 2014)

Tree species	Basic wood density	Biomass expansion factor	Root/Shoot ratio
*Pinus densiflora*	0.472	1.413	1.254
*Pinus koraiensis*	0.408	1.812	1.283
*Larix kaempferi*	0.453	1.335	1.291
*Quercus variabilis*	0.721	1.338	1.324
*Quercus mongolica*	0.663	1.603	1.388
Mixed forest 1 *(Pinus densiflora, Quercus variabilis)*	0.5965	1.375	1.289
Mixed forest 2 *(Pinus densiflora, Quercus mongolica)*	0.5675	1.508	1.321

5 $${\mathrm{C }}_{{t}_{i}}= {\mathrm{V}}_{{t}_{i}}\times {\mathrm{D}}_{j}\times {\mathrm{BEF}}_{j}\times \left(1+{\mathrm{RS}}_{j}\right)\times \mathrm{CF}$$

6 $$\Delta {\mathrm{CS}}_{{t}_{i}} =\frac{{CS}_{{t}_{i+1}}-{CS}_{{t}_{i}}}{{t}_{i+1}-{t}_{i}}$$

7 $${{\Delta \mathrm{CO}}_{2}}_{{t}_{i}}= \Delta {\mathrm{CS}}_{{t}_{i}}\times \frac{44}{12}$$

where *C* is carbon stock (tC/ha), *V* is the stand volume (m^3^/ha), *D* is the basic density of the wood, BEF is called biomass expansion factor, *RS* is the root to shoot ratio, *CF* is the carbon fraction (biomass to carbon, IPCC default = 0.5), ${t}_{i}$ is the year, *j* is tree species, ${CS}_{{t}_{i}}$ and ${CS}_{{t}_{i+1}}$ are the annual change in carbon stock (tC/ha/year) calculated at ${t}_{i}$ and ${t}_{i+1}$ year, ${{\Delta CO}_{2}}_{{t}_{i}}$ is annual carbon dioxide sequestration (tCO_2_/ha/year), and 44/12 is the stoichiometric ratio of CO_2_ and C.

#### Application scenario

In this study, three time periods were used: the current base period (2010–2015), 2030 (2026–2035), and 2050 (2046–2055). The analysis reflects the current transition in the forest physiognomy in Republic of Korea from young-matured stands to over-mature forests. Four scenarios were established individually according to the application of the variables which are climate change and forest management intensity in South Korea [41].

Under each scenario, the cost of adapting various management strategies to future climate change was estimated. Also, these scenarios can be analyzed chronologically. The four scenarios were finally summarized as follows (Table 3).

**Table 3:** Scenario classification for this study

Scenario	Description
1	Current climate maintenance (no climate change occurs) Overprotection
2	Future climate change scenario RCP 8.5 Overprotection
3	Future climate change scenario RCP 8.5 Current level forest management: • Clear-cut harvest according to the legal final cutting age (~ 15,000 ha year^−1^) • Harvest by thinning (~ 30%) Reforestation
4	Future climate change scenario RCP 8.5 Ideal level of forest management: • Clear-cut harvest according to the legal final cutting age (~ 35,000 ha year^−1^) • Harvest by thinning (~ 30%) Reforestation

In Scenario 1, climate change was not considered, which means the current climate condition is maintained. Also, an overprotective forest was assumed that no forest management, such as harvest, is performed during study periods.

In scenarios 2, forest changes were predicted by applying the IPCC representative concentration pathway (RCP) scenario 8.5. RCP 8.5 is a scenario (business as usual BAU) in which greenhouse gases are emitted without reduction of greenhouse gases and is mainly used to predict changes in forests due to climate change. Therefore, scenario 2 reflected the climate change and the overprotective forest which is same as scenario 1.

In scenario 3, climate change was considered like scenario 2, but it includes climate change adaptation strategies. It applied the current forest management practices of clear-cut harvest, in accordance with the legal final cutting age, harvest by crown thinning, and reforestation. According to the statistics of the Korea Forest Service, current annual forest tending area is approximately 130,000 ha, of which the legal final cut is approximately 15,000 ha and thinning is 30%, which is conducted twice a year [51]. Therefore, it was set to implement thinning for 20 and 40 years of age, and density management by 30% crown thinning in the selected rank in order of highest to lowest according to the stand volume among the age [52]. Based on the Korean policy about the cutting age, the cutting ages of red Pine, Korean pine, Japanese larch, cork oak, Mongolian oak, mixed forest A, mixed forest B were 60, 60, 50, 60, 60, 60 and 60 respectively [53]. At this time, it was set that the major tree species which can properly grow according to climate change are planted during reforestation in the clear-cut area according to normal final age [42, 54]. These management level were continuously maintained under Scenario 3.

Scenario 4 was based on the premise that ideal forest management would be implemented, which would cover all the above scenarios; the area of clear-cut harvest was approximately 35,000 ha/yr using the legal final cutting age, harvesting by crown thinning, and the use of appropriate tree species for reforestation. Under the Korea Forest Service forest tending promotion plan (2018–2037), 200,000 ha of cutting (legal cut, thinning, etc.) is planned annually [55]. Therefore, in this study, the thinning intensity, the frequency of implementation for which the current state was maintained, and the addition of 35,000 ha of legal final cut to maximize forest growth were applied to Scenario 4 [41, 56].

In short, scenario 2, 3, and 4 modeled the impact of climate changes based on RCP 8.5 with different intensity of forest managements based on the adaptation strategies. When this study applied the forest management, the spatial area was classified into two types of management forest areas and restricted forest areas [39]. Based on forest regulations and spatial data, the harvest and management area of the forest occupied 3,178,900 ha, approximately 52.48% of the total forest. Furthermore, Korea Forest Services suggested and authorized clear-cut harvest for each tree species according to the final age of maturity [57]. Therefore, the cycle of final felling and regeneration is according to the tree species in the manageable area. In addition, we did not include any natural disturbances and changes in land cover such as afforestation or deforestation in the simulation periods.

### Data preparation and modifications

To quantitatively understand the effects of climate change, a baseline climate scenario and climate change scenario were used in this investigation. For the current climate assessment, the average temperature data from 2000 to 2010 were considered as the fixed baseline climate. For future climate changes, the realistic climate scenario (Hadgem3ra–RCP8.5) was utilized, assuming business-as-usual and no reduction in global warming [58–62].

A forest-type map and Korean National Forest Inventory (NFI) program data were used to construct a basic dataset for model simulation. The dataset is based on spatial data of 1 km $\times$ 1 km grid cell, and it was constructed with the area of about 60,564 km^2^ (6,056,400 ha) only for stocked forest land. The forest-type map provides information such as forest physiognomy, tree species, DBH class, age class, and crown density of the stand. These basic data were created using aerial photographs from 2009 to 2013 at a scale of 1:15,000 and was applied to the modeling at a scale of 1:5,000 [63]. The NFI includes circulating systematic sampling survey over five year periods, 20% each year (i.e., 800 plots from among 4000 permanent plots on a 4 km × 4 km grid) of all South Korean forests [64, 65]. Four circular sample plots were located at the intersections of 4 km $\times$ 4 km grid lines. Forest field survey provided data reflecting the characteristics of the forest. The measured forest dataset (DBH, height, age, tree species, and stand volume) allowed stand categorization, and this was combined with a site investigation dataset (coordinates, quality of locality, elevation, slope, and aspect) [66]. In this study, the 5^th^ NFI (2006–2010) was the basis and was supplemented with 6th NFI (2011–2015) data [27]. The representative tree species and baseline stand volumes were established by combining the spatial and attribute data from the forest and site datasets [67, 68]. The site index of each stand was estimated from the height information of the dominant tree, according to the field survey NFI data, and applied to the model [28, 69, 70].

For forest management modeling, it was assumed that the forest physiognomy was confirmed, when reaching the legal cutting age [71], and forest physiognomy with a higher age class and stand volume was selected first. According to the sustainable forest management law established by the Korea Forest Service and the plan for effective thinning at the Korea National Institute of Forest Science, the Korea forest cover needs to be thinned out by 30% at a stand age of 20–40 years [16, 72]. This study adopted this legal requirement. To calculate the appropriate budget for the forest sector, considering future climate change, it was assumed that areas were vulnerable to reduced forest growth, and the management areas were then identified using spatiotemporal analysis on a 1 km × 1 km raster derived from the forest type map. The budget for cutting and reforestation, for each tree species, was thus calculated in grid form. The per grid costs of future forest management were calculated by reviewing the reforestation budgets currently implemented in the Republic of Korea [52] (Table 4).

**Table 4:** Forest sector budget unit prices (Korea Forest Service, 2018)

Forest tending works design, audit review, and business execution guidelines Unit: KRW/ha (USD/ha)				
Mowing and vine removal	Tending of young growth	Reforestation (pruning + thinning + installation and selection of working roads)	Collecting of products	Basic design implementation and design supervision
1,370,968 (1,142)	1,153,379 (961)	1,453,936 (1,212)	17,947,395 (14,956)	127,321 (106)

Felling and bucking costs and primary transport cost Personnel expense + sawing machine expense + gas and oil expenses = 252,696 KRW/day (USD/day)					
Division	*Pinus densiflora*	*Pinus koraiensis*	*Larix kaempferi*	Other coniferous forests	Other broad-leaved forests
Felling and bucking cost KRW/m^3^ (USD/m^3^)	18,622 (16)	16,356 (14)	19,246 (16)	27,084 (23)	20,747 (17)
Primary transportation cost in mountainous KRW/m^3^ (USD/m^3^)	21,939 (18)	20,508 (17)	20,508 (17)	20,508 (17)	21,939 (18)

Reforestation unit price of major species of trees KRW/ha (USD/ha)				
*Pinus* *densiflora*	*Pinus koraiensis*	*Larix* *kaempferi*	*Quercus variabilis*	*Quercus mongolica*
6,176,827 (5147)	6,474,693 (5396)	6,072,753 (5061)	6,288,078 (5240)	7,269,601 (6058)

## Results

### Growth of forest trees

**Fig. 3:**
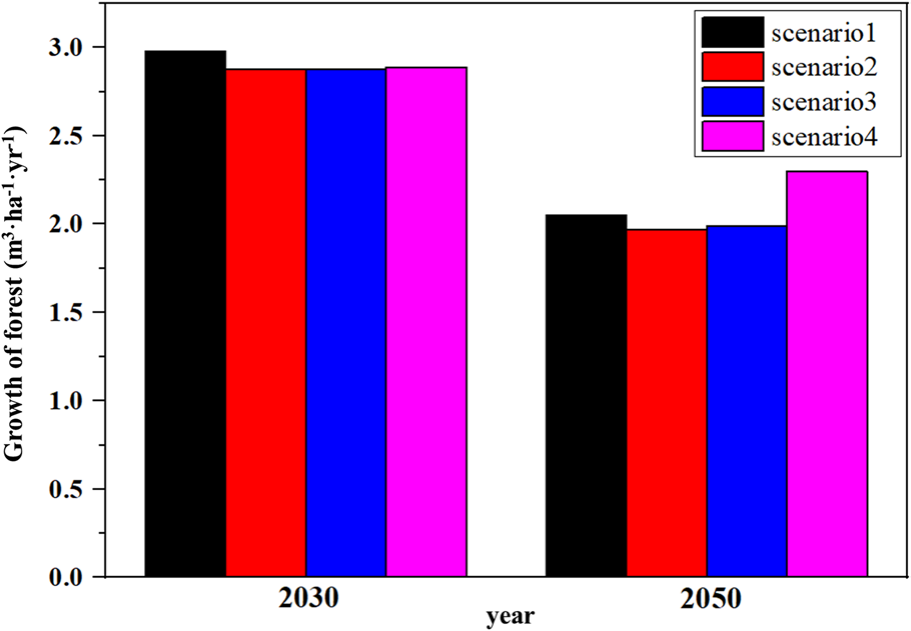
Forest growth in the 2030s and 2050s by scenario

**Fig. 4:**
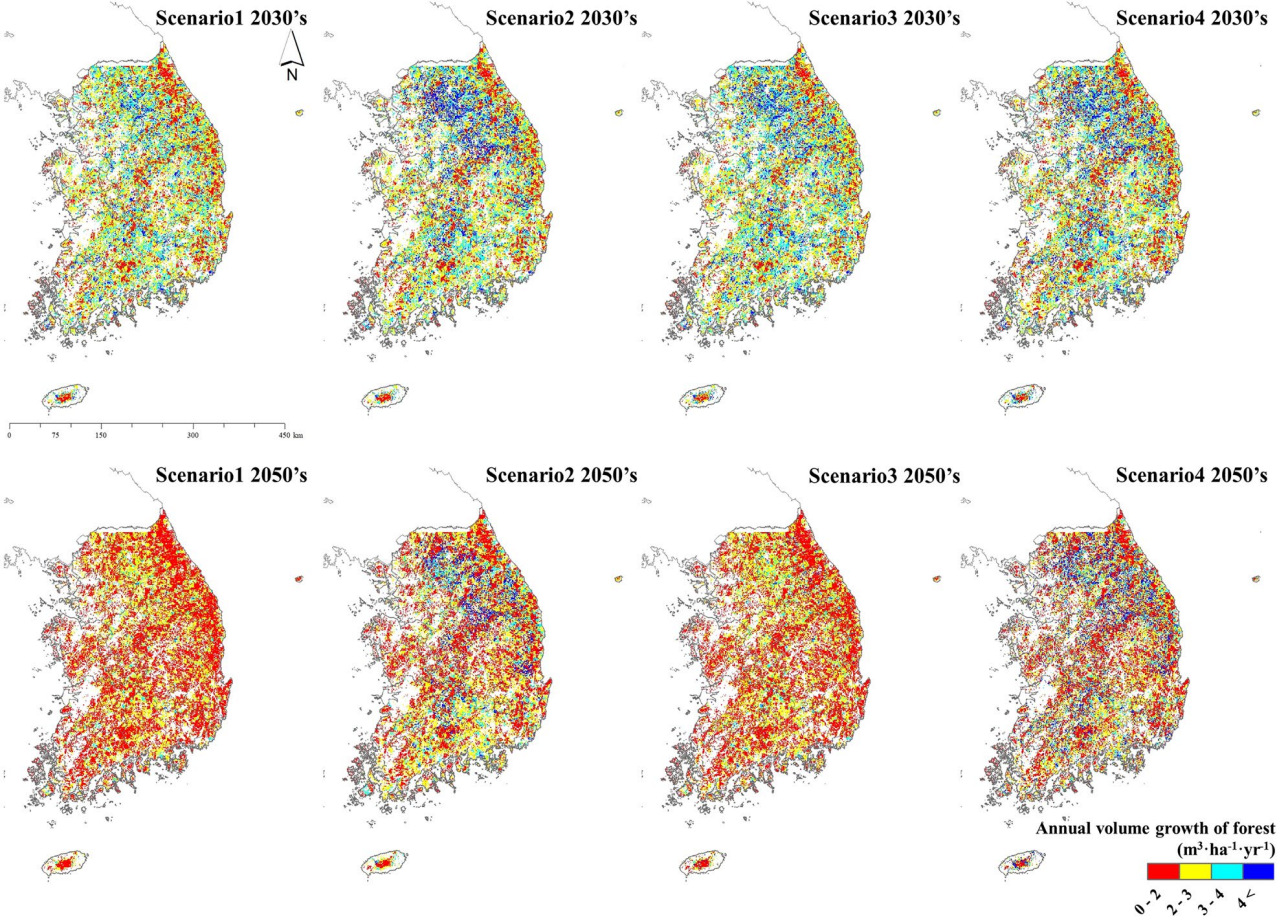
Estimated distribution of forest tree growth in South Korea. Scenario 1: current climate maintenance; Scenario 2: climate change; Scenario 3: current level of forest management; Scenario 4: ideal level of forest management based on future spatiotemporal forest growth maps for the 2030s and 2050s

Using the KO-G-Dynamic model, an annual forest growing stock volume was estimated for each scenario, and the annual growth results were analyzed. The results were derived using different methods, depending on the scenario-specific options. The study focused on years 2030 and 2050 to match NDC milestones. In the 2030s, the annual growth rates under scenarios 1, 2, 3, and 4 were 2.98 m^3^ ha^−1^, 2.88 m^3^ ha^−1^, 2.88 m^3^ ha^−1^, and 2.89 m^3^ ha^−1^, respectively. The annual growth rates for scenarios 1, 2, 3, and 4 in the 2050s were 2.05 m^3^ ha^−1^, 1.97 m^3^ ha^−1^, 1.99 m^3^ ha^−1^, and 2.30 m^3^ ha^−1^, respectively (Fig. 3). Although the accumulation was higher in the 2050s than in the 2030s, annual growth relatively decreased. The processes of slashing, felling, cutting, and thinning decreased annual growth rates; however, growth thereafter significantly increased when compared to scenarios without management (Fig. 4).

### Amount of CO_2_ sequestration in the forests

**Fig. 5:**
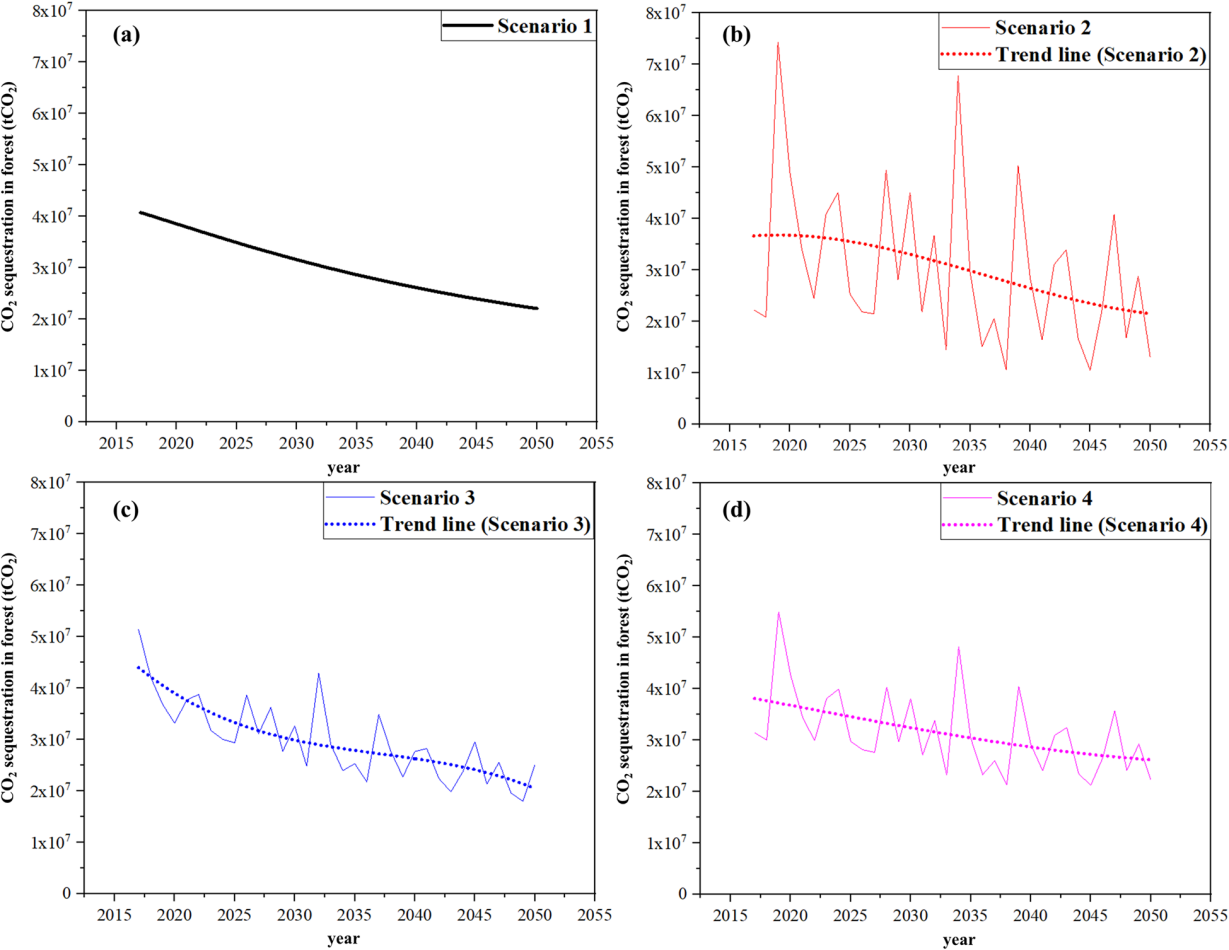
Time series (2017–2050) of forest CO_2_ sequestration showing annual amount of CO_2_ sequestration (tCO_2_ year^−1^) forecast under each scenario, considering climate change and forest management: **a** CO_2_ sequestration when the current climate is maintained and forests are not managed; **b** CO_2_ sequestration when the climate changes and forests are not managed; **c** CO_2_ sequestration when climate changes and current forest management are maintained. **d** CO_2_ sequestration when climate changes and an ideal forest management scenario that maximizes forest growth are applied

**Fig. 6:**
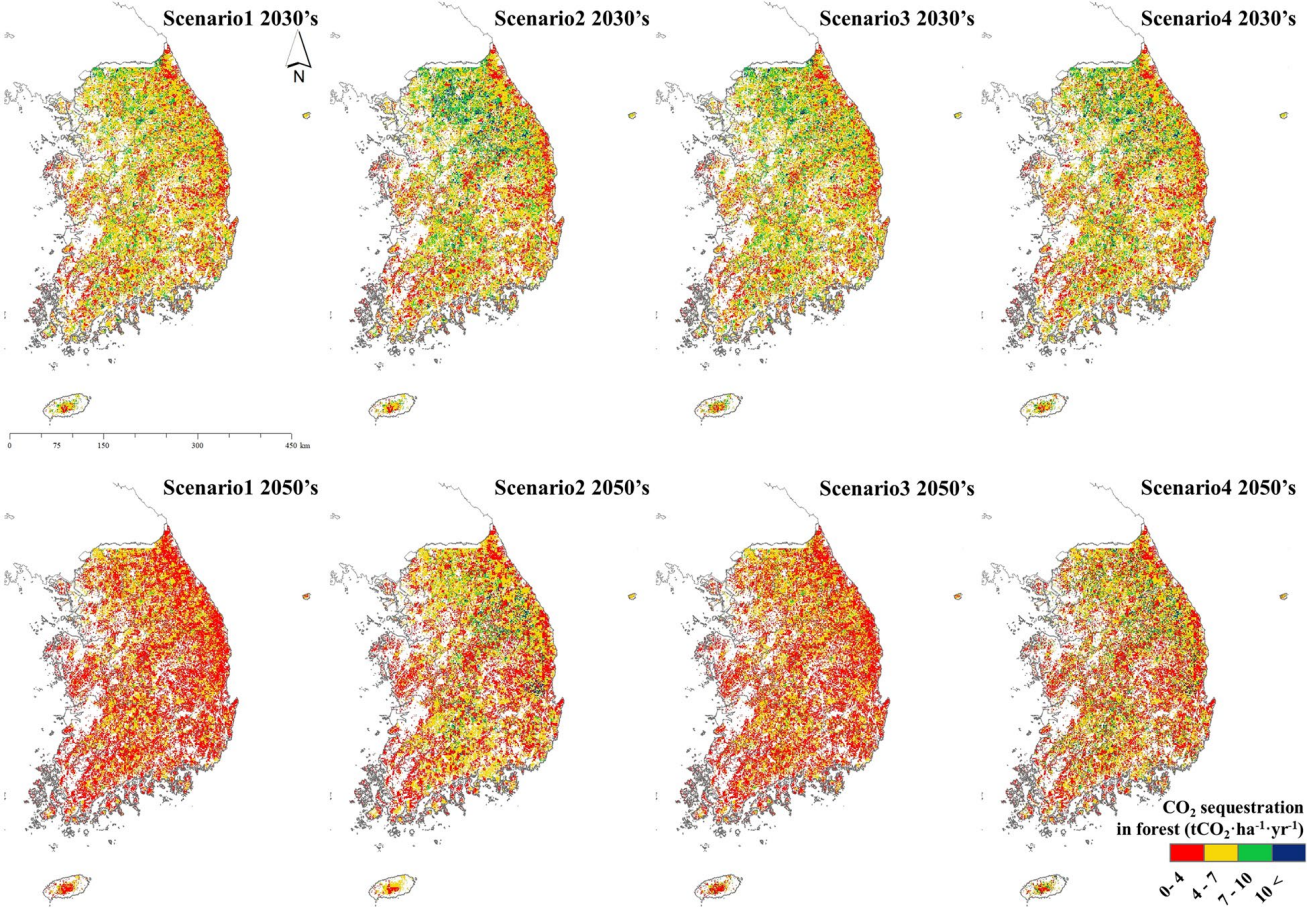
Distribution of estimated CO_2_ sequestration in South Korean forests, according to the climate change and forest management scenarios for the 2030s and 2050s

The amount of CO_2_ sequestration was calculated based on the forest growing stock volume calculated above. In 2030s, the amounts of CO_2_ sequestration for scenarios 1, 2, 3, and 4 were estimated as 34,611,300 tCO_2_ year^−1^, 33,556,800 tCO_2_ year^−1^, 32,419,236 tCO_2_ year^−1^, and 33,598,573 tCO_2_ year^−1^, respectively. For 2050s, the amounts of CO_2_ sequestration for scenarios 1, 2, 3, and 4 were 23,784,800 tCO_2_ year^−1^, 23,038,500 tCO_2_ year^−1^, 23,355,321 tCO_2_ year^−1^, and 27,001,520 tCO_2_ year^−1^ (Fig. 5), respectively. All scenarios had lower absorption levels in the 2050s than in the 2030s (Fig. 6). The forest management scenarios (3 and 4) absorbed more carbon than those without management. This is the result of improving South Korea's unbalanced age class structure through forest management from a long-term perspective. The simulations in this study did not include CO_2_ emissions losses during harvesting. The analysis of CO_2_ sequestration levels exhibited variation according to tree species and forest growing stock volume.

### Cost–benefit analysis of forest management

The costs and benefits of each scenario were then analyzed. In Korea, although the proportion of private forests is higher than that of national and public forests, the government emphasizes the value of forests as a public property and notifies forest owners about the government's laws and policies as part of their governance [73]. Therefore, in this study, the costs were calculated according to the forestry unit price, which was estimated from the budget of the Korea Forest Service [74], and the benefits were estimated according to the transactional economic gains from log trading and CO_2_ sequestration [75].

The annual costs for scenarios 1, 2, 3, and 4 in 2030 were 321 billion KRW (267 million USD), 418 billion KRW (348 million USD), 639 billion KRW (533 million USD), and 722 billion KRW (602 million USD), respectively, whereas those in 2050 were 321, 453, 506, and 618 billion KRW (267, 377, 421, and 516 million USD), respectively (Table 5). In Scenario 4, which had the most sustainable results, the cost of thinning, including other processes such as cleaning, cutting, mowing, and vine removal, was 521 billion KRW (434 million USD) per year in the 2030s. In the 2050s, the cost of thinning was 397 billion KRW (331 million USD) per year. The cost of the clear-cut harvest to the legal final cutting limit based on the spatial simulation of the unit price per tree species was estimated to be 1.1 billion KRW (0.9 million USD) in 2030 and 1.2 billion KRW (1 million USD) in 2050. According to Korean forestry statistics, the percentage of collecting product by forest yarding, in forest tending work, is approximately 30% [76, 77]. Collecting products by forest yarding is the process of cutting, using forestry machines, and yarding and transporting both wood and any by-products that have value as biomass [24]. The estimated cost was therefore calculated by applying the above percentage of collected yarding product in the context of future environmental and policy situations. In the case of reforestation, the appropriate species were identified, recognizing the influence of climate change, and the unit cost for reforestation of that tree species was applied. According to the legal final cutting age, the cost of reforestation with suitable tree species, when considering climate change in the cut over area, was 192 billion KRW (160 million USD) in 2030 and 211 billion KRW (176 million USD) in 2050. Other costs, such as gas, oil, and personnel expenses, were applied to the area values for thinning and final cutting.

**Table 5:** Cost analysis for forest scenarios 1–4 (in units of 100 million KRW, in units of million USD). Sustainable scenarios add forest management prescriptions and budget requirements

Cost comparison based on climate change	Scenario 1		Scenario 2		Cost comparison based on the level of forest management	Scenario 3		Scenario 4	
Classification	2030	2050	2030 (15 years cumulative)	2050 (35 years cumulative)	Classification	2030 (15 years cumulative)	2050 (35 years cumulative)	2030 (15 years cumulative)	2050 (35 years cumulative)
(A) FMOACB KRW/year (USD/year)	2,090 (174)	2,090 (174)	2,090 (174)	2,090 (174)	(A’) HTnCuA (ha)	2,474,000	4,402,900	2,474,000	4,402,900
(B) REFACB KRW/year (USD/year)	1,122 (93)	1,122 (93)	1,122 (93)	1,122 (93)	(B’) HTnCP KRW/year/ha (USD/ year/ha)	0.0316 (0.000002)	0.0316 (0.000002)	0.0316 (0.000002)	0.0316 (0.000002)
Total cost of forest resources management KRW/year (USD/year) [(A) + (B)]	3,212 (267)	3,212 (267)	3,212 (267)	3,212 (267)	Total annual cost due to harvest by thinning KRW/year (USD/year) [(A’) * (B’) /Accumulated year]	5,217 (434)	3,979 (331)	5,217 (434)	3,979 (331)
(C) Vulnerability cumulative area VCuA (ha)			6,637,600	21,154,400	(C’) CC.LEFA.CuA (ha)	225,000	525,000	446,400	1,146,400
(D) AvCPVM KRW/year (USD/year)			0.0021 (0.000182)	0.0021 (0.000182)	(D’) CC.LEFA PGSAv.CuA (m^3^/year)	150.67	147.30	192.61	203.19
(E) CVMAP KRW/year (USD/year) [(C) * (D) /Accumulated year]			968.6 (80.7)	1,323 (110)	(E’) AvCD.SP.GS /TiL.CC.LEFA KRW/m^3^/year (USD/m^3^/year)	0.00000197 (0.0000000001)	0.000002 (0.0000000001)	0.00000192 (0.0000000001)	0.00000194 (0.0000000001)
					(F’) CC.GS.LEFA.SP KRW/year (USD/year) [(C’) * (D’) * (E’)/Accumulated year]	4.472 (0.372)	4.430 (0.369)	11.03 (0.919)	12.91 (1.076)
					(G’) Av.OE.LOG KRW/year/ha (USD/year/ha)	0.0021 (0.000015)	0.0021 (0.000015)	0.0021 (0.000015)	0.0021 (0.000015)
					(H’) C.LOG.AP KRW/year (USD/year) [(C’) * (G’) /Accumulated year]	32.83 (2.736)	32.83 (2.736)	65.14 (5.428)	71.69 (5.974)
					(I’) ColPCAv KRW/year/ha (USD/year/ha)	0.0003 (0.000000023)	0.0003 (0.000000022)	0.0003(0.000000026)	0.0004 (0.000000029)
					(J’) ColPC.LOG KRW/year (USD/year) [(C’/ Accumulated year) * (I’)] * 0.3	1.462 (0.121)	1.441 (0.120)	3.343 (0.278)	4.164 (0.347)
					(K’) DSP.AvREF KRW/year/ha (USD/year/ha)	0.0762 (0.0000052)	0.0696 (0.0000048)	0.0647 (0.0000045)	0.0646 (0.0000044)
					(L’) CREF.AcDSP.LOG KRW/year (USD/year) [(C’) * (K’) /Accumulated year]	1,143 (95)	1,044 (87)	1,926 (160)	2,117 (176)
Total potential cost KRW/year (USD/year)	3,212 (267)	3,212 (267)	4,181 (348)	4,535 (377)	Total potential cost KRW/year (USD/year)	6,399 (533)	5,062 (421)	7,223 (602)	6,185 (516)

^(A)^FMOACB: Forest management operation applied to the current budget; ^(B)^REFACB: Reforestation applied to the current budget (e.g., forest tending works, mowing, vine removal, collecting products, basic design implementation, and design supervision); ^(C)^VCuA: Vulnerable, cumulative area; ^(D)^ AvCPVM: Average costs spent to the point of vulnerability management; ^(E)^CVMAP: Cost of vulnerability management area consumed up to that point

^(A’)^HTnCuA: Harvest by thinning, cumulative area; ^(B’)^HTnCP: Harvest by thinning, costs consumed by that point; ^(C’)^CC.LEFA.CuA: Clear-cut harvest according to the legal final cutting age acquired cumulative area; ^(D’)^CC.LEFA.PGSAv.CuA: Clear-cut harvest according to the legal final cutting age point and forest growing stock volume average in the cumulative area; ^(E’)^AvCD.SP.GS./TiL.CC.LEFA: The average of the costs that are generated differently according to the tree species and forest growing stock volume at the time of implementation in the clear-cut harvest according to the legal final cutting age; ^(F’)^CC.GS.LEFA.SP: Cutting costs according to the forest growing stock volume, that reached the legal final cutting age, by tree species; ^(G’)^Av.OE.LOG: The average of other expenditures in addition to the harvest logging (e.g., personnel, sawing machine, gas, and oil expenses); ^(H’)^C.LOG.AP: The cost of the harvested logging area consumed up to that point (e.g., personnel, sawing machine, gas, and oil expenses); ^(I’)^ColPCAv: Collected products by forest yarding cost average; ^(J’)^ColPC.LOG: Collected product costs at harvest logging; ^(K’)^DSP.AvREF: Considering the differences in tree species, the average reforestation cost; ^(L’)^CREF.AcDSP.LOG: The cost of reforestation, taking into account differences in tree species, according to harvest logging

**Fig. 7:**
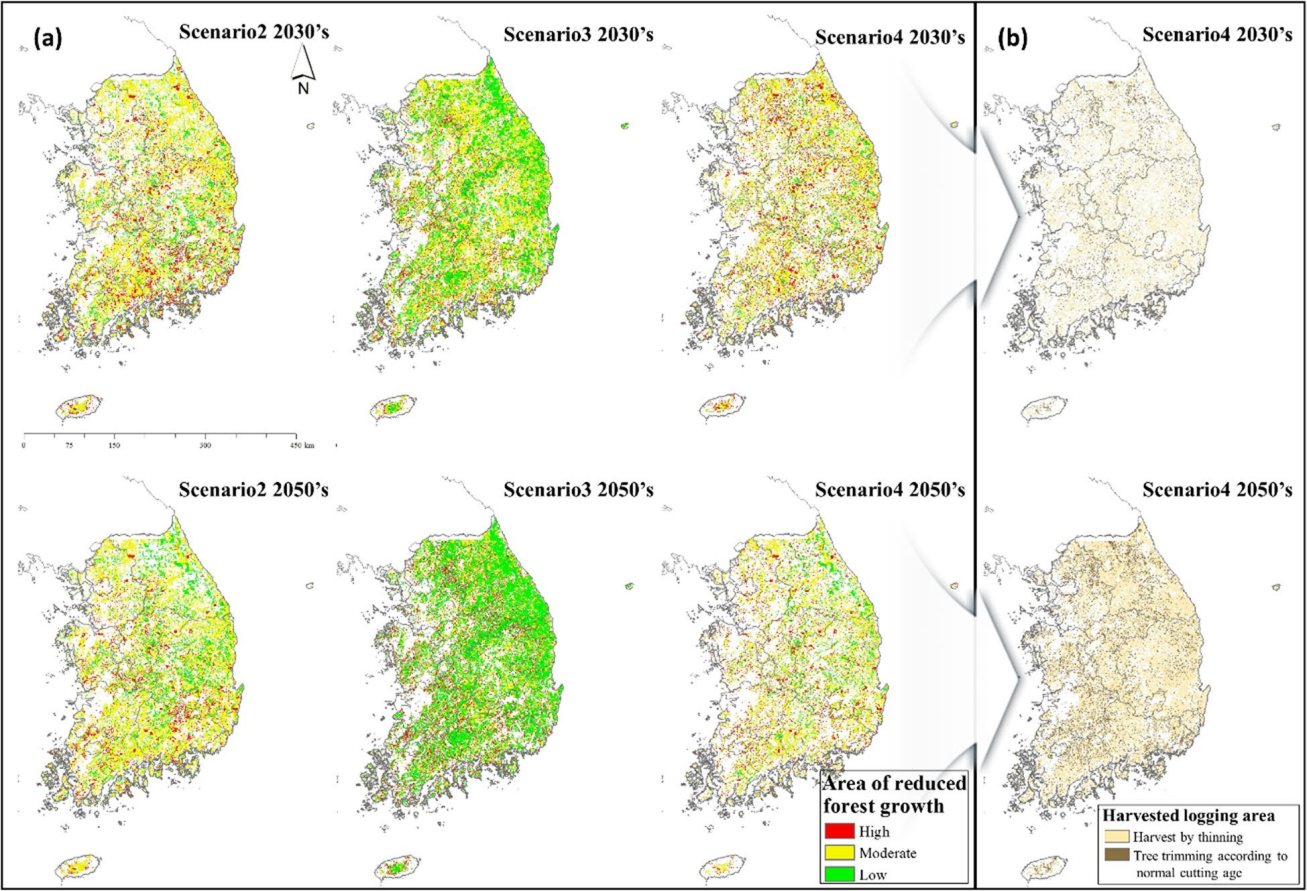
Sensitive areas and direction of management in the forest. **a** Sensitivity grading and distribution maps identifying the areas where future forest growth can be expected to be reduced when compared to the current climate conditions. **b** Forest scenario for the cumulative implementation area in Scenario 4, which had ideal conditions with optimal growth and CO_2_ sequestration

The estimated high forest management expenditure in 2030 reflects the high initial economic cost. However, forest growth is reduced by the degree of forest sensitivity to climate change, and the corresponding management area is decreased (Fig. 7).

**Fig. 8:**
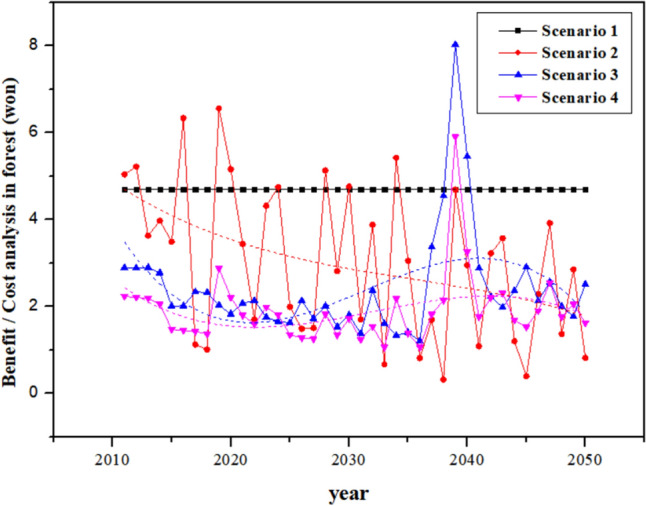
Cost–benefit analysis. Estimation of the benefits relative to the costs by scenario. Trend analysis of the estimated cost-benefits for different forest types. Scenario 1 exhibits a trend in which the cost–benefit value is maintained as it is based on the current climate and no forest management; scenarios 2–4 assume climate (Hadem3ra–RCP8.5) variability and different degrees of forest management. The tendencies of scenarios 2–4 was estimated through trend lines

The benefits of each scenario were estimated from the above in conjunction with the log trading price suggested by the Korea Forest Service and the carbon credit price (KOC: Korea Offset Credit) in 2019 [78]. The benefits for scenarios 1, 2, 3, and 4 in 2030 were 1,508 billion KRW (1,257 million USD), 1,275 billion KRW (1,063 million USD), 1,237 billion KRW (1,024 million USD), and 1,207 billion KRW (995 million USD), respectively, whereas those in 2050 were 1,508 billion KRW (1,257 million USD), 1,057 billion KRW (881 million USD), 1,040 billion KRW (855 million USD), and 1,088 billion KRW (890 million USD), respectively (Table 6). It has been shown that more benefits are derived from a long-term than from a short-term perspective (Fig. 8).

**Table 6:** Analysis of benefits generated in 2030 and 2050 by scenario (in units of 100 million KRW, in units of million USD)

Classification	Scenario 1		Scenario 2		Scenario 3		Scenario 4	
	2030 (15 years cumulative)	2050 (35 years cumulative)	2030 (15 years cumulative)	2050 (35 years cumulative)	2030 (15 years cumulative)	2050 (35 years cumulative)	2030 (15 years cumulative)	2050 (35 years cumulative)
Carbon trading revenue from forest CO_2_ sequestration KRW/year (USD/year) [(Carbon credit price * CO_2_ sequestration in forest)/Accumulated year]	15,085 (1257)	15,085 (1257)	12,759 (1063)	10,577 (881)	12,288 (1024)	10,262 (855)	11,945 (995)	10,686 (890)
Log trading revenue from tree trimming KRW/year (USD/year) [(Volume * Log trading price by tree species)/Accumulated year]					87 (7)	143 (11)	131 (10)	200 (16)
Total potential benefit KRW/year (USD/year)	15,085 (1257)	15,085 (1257)	12,759 (1063)	10,577 (881)	12,376 (1031)	10,406 (867)	12,077 (1006)	10,887 (907)

## Discussion

Using forests for CO_2_ reduction has become a direct method in achieving the NDC. This study identified the latest forest policy trends, including the trading values of wood and carbon, and derived an optimal forest management plan. Scenario 1 was a hypothetical potential future without climate change and harvest, Scenario 2 incorporated climate change but overprotective forest, and scenarios 3 and 4 included climate change and management. Although the cost–benefit ratios for scenarios 2, 3, and 4 were similar, the carbon sequestration was considerably higher in the management scenarios. This indicates that it would be better to change the current policy from passive to more active forest management, as it will have the same cost–benefit ratio with more carbon sequestration, which is in line with the Paris Agreement.

The optimized forest management scenario, which encourages greater growth than current forest practices and which accommodated the changing climate, was advantageous in terms of the amount of CO_2_ sequestered and economic feasibility [79]. In the 2030s, the optimal forest management scenario includes prevention of young-matured stands from becoming over-mature forest. However, in the short term, the estimated 2.89 m^3^ ha^−1^ of forest growth is smaller than the forest growth of 2.98 m^3^ ha^−1^ under current management. Contrastingly, in this scenario in the 2050s, the forest growth under optimal management was on average 0.4 m^3^ ha^−1^ greater than that in the non-management climate change scenario, demonstrating the meaningful outcomes of continuous management practices. Previous investigations have also shown this to be a more effective long-term forest management strategy [80–82].

In many previous studies in South Korea, only one forest management method, such as cutting or thinning, was generally considered, and mixed forest management was rarely addressed. Moreover, studies on forest management have predominantly focused on specific tree species (e.g., *Pinus densiflora*, *Pinus koraiensis*, and *Larix kaempferi*.), and studies on forest physiognomy as a whole are rare [47, 83–87]. Furthermore, there have been few studies on how forest management measures can increase adaptability to climate change [88, 89]. Therefore, this study not only reinforces our knowledge of adaptive capacity but also suggests the necessary budget for appropriate forest management measures, based on the mechanisms of forest physiognomy that are increasingly sensitive to climate change. However, the study did not consider dead organic matter, soil carbon pools, and harvested wood product, which Korea considers in reporting NIR (National Inventory Report) currently [90]. In addition, the actual natural (disease and insect pests, wind damage, landslide) and anthropogenic (forest fires, illegal activities, land cover change) disturbances that occur every year should be more considered to develop further forest modeling. In future research, it is necessary to find advanced strategies in conjunction with domestic dead organic matter, soil carbon pools and global forest management models reflecting the actual dynamic of forestland [16, 52, 91, 92].

Sequestration based on forest growth according to the improvement of the age class structure through forest management is a key contributor in achieving NDC [93]. Therefore, in this study, a forest growth model was used to analyze whether sequestration based on growth could contribute to the achievement of the NDC. Evidently, under climate change, the Republic of Korea’s forest sector could achieve its NDC target of 26 million tons of CO_2_ sequestration by 2030. Indeed, it is estimated that up to 30 million tons could be absorbed if the forest growth sector was managed well. These results demonstrate that forest management is more important for the promotion of CO_2_ sequestration than other management measures, especially when considering the forest growth due to climate change. Moreover, the analysis results of the sensitive areas, where forest growth may decrease with climate change, showed that forest management reduces the sensitive area by approximately 1,500,000 ha compared to climate change scenarios without such management.

According to the Korea Forest Service budget, the annual cost of forest tending works is 209 billion KRW (174 million USD), and the cost of reforestation is 112.2 billion KRW (93 million USD) [94]. Furthermore, only 5% of the deforestation amount was applied in collecting products [95]. However, this budget might be insufficient when considering climate adaptation, promotion of forest health, and reduction of disaster risk in conditions of extreme climate changes. Therefore, sustainable forest management plans, developed using the KO-G-Dynamic model, should be implemented for areas where forest growth can decrease the risk imposed by climate change. In this way, policymakers can better understand the future of forest area in terms of time and space. Furthermore, forest tending works and reforestation unit costs per species can be estimated. At this time, the required forest management costs, for the recommended forest tending and reforestation, are approximately triple the current budget [96]. Therefore, the development and application of appropriate modeling can help in the assessment of current sensitivity and provide direction for achieving the NDC.

The present results also contribute to national sustainable development goals (SDGs). In the Republic of Korea, the forest sector plays an important part in achieving national SDGs, especially in aspects of Goal 15 and with close links to Goal 13 when considering NDCs [97]. As this study indicates, forest management is the key to GHG reduction, and application of an integrated forest model allows the quantification of diverse goals. This is important in understanding how the forest ecosystem is affected by climate change and how social, environmental, and economic factors can be changed by related adaptive policy instruments [98, 99]. The quantified results from the KO-G-Dynamic model can be used by decision makers to promote future policy assessments and by forest landowners who face climate change [100]. Candidate policies can be tested prior to forest activities and plans can be developed to help maximize carbon sequestration, as well as income [101]. Such future blueprints can be created using integrated modeling and provide policy-oriented solutions focusing on the NDCs and SDGs in South Korea [102].

## Conclusions

In this study, the KO-G-Dynamic model, a spatiotemporal forest growth model, was used to analyze forest management strategies, including costs and benefits, in response to climate change and commitments to GHG reductions. A number of forest management scenarios were simulated using a KO-G-Dynamic model, based on forest growth and the role of forests as a carbon sink, and an optimal management scenario was identified. Increased forest growth leads to an increase in CO_2_ sequestration, contributing in the achievement of the NDC, and is also linked to the SDGs. In the long-term assessment, climate change increased the likelihood of reduced forest growth and required increased budgets as the areas of vulnerability increased. The forest sector’s NDC target for South Korea, which was considered a difficult goal, was possible if appropriate forest management practices were implemented. With the arrival of the “new climate change regime,” the nation’s forest management policy needs to operate with clear goals and directions. Therefore, it is important to quantify the GHG contributions of forests under sustainable management practices and accordingly tailor an appropriate budget. However, it is also essential to establish and improve laws and systems such as the Carbon Absorption Promotion Act and the Greenhouse Gas Reduction Act, as they can be used to manage and implement appropriate practices.

## Data Availability

Forest data are available online through the Forest Big Data Exchange of the South Korea website (https://www.bigdata-forest.kr/). Climate data were obtained from the Korea Meteorological Administration (http://www.climate.go.kr/home/CCS/contents_new/35_download.php). The estimation of forest growth in units of 1 km, according to climate change using the KO-G-Dynamic model, can be downloaded from the Korea Adaptation Center for Climate Change (http://motive.kei.re.kr/).
